# Comparison of Non-Oncological Postoperative Outcomes Following Robotic and Laparoscopic Colorectal Resection for Colorectal Malignancy: A Systematic Review and Meta-Analysis

**DOI:** 10.7759/cureus.27015

**Published:** 2022-07-19

**Authors:** Chetna Ravindra, Emmanuelar O Igweonu-Nwakile, Safina Ali, Salomi Paul, Shreyas Yakkali, Sneha Teresa Selvin, Sonu Thomas, Viktoriya Bikeyeva, Ahmed Abdullah, Aleksandra Radivojevic, Anas A Abu Jad, Anvesh Ravanavena, Prachi Balani

**Affiliations:** 1 General Surgery, California Institute of Behavioral Neurosciences & Psychology, Fairfield, USA; 2 Internal Medicine, California Institute of Behavioral Neurosciences & Psychology, Fairfield, USA; 3 Behavioral Neurosciences and Psychology, California Institute of Behavioral Neurosciences & Psychology, Fairfield, USA

**Keywords:** robotic colorectal surgery, laparoscopic colorectal surgery, postoperative outcomes, minimal access surgery, colorectal cancer

## Abstract

The objective of this systematic review and meta-analysis is to compare the postoperative outcomes of robotic and laparoscopic colorectal resection for colorectal malignancy. We performed a systematic review using a comprehensive search strategy on several electronic databases (PubMed, PubMed Central, Medline, and Google Scholar) in April 2022. Postoperative outcomes of robotic versus laparoscopic surgery for colorectal cancer were compared using 12 end points. Observational studies, randomized controlled trials, and nonrandomized clinical trials comparing robotic and laparoscopic resection for colorectal cancer were included. The statistical analysis was performed using the risk ratio (RR) for categorical variables and the standardized mean differences (SMD) for continuous variables. Sixteen studies involving 2,318 patients were included. The difference in length of hospital stay was significantly shorter with robotic access (SMD = -0.10, 95% CI = -0.19, -0.01, P = 0.04, I^2 ^= 0%). Regarding intra-abdominal abscesses, the analysis showed an advantage in favor of the robotic group, but the result was not statically significant (RR = 0.54, 95% CI = 0.28, 1.05, P = 0.07, I^2^ = 0%). Mechanical obstruction was found to be higher in robotic group, favoring laparoscopic access, but was not significant (RR = 1.91, 95% CI = 0.95, 3.83, P = 0.07, I^2^ = 0%). There was no difference in time to pass flatus and consume a soft diet. The rates of anastomotic leakage, ileus, wound infection, readmission, mortality, and incisional hernias were similar with both approaches. Robotic surgery for colorectal cancer is associated with a shorter hospital stay, with no differences in mortality and postoperative morbidity.

## Introduction and background

Colorectal cancer is the third most common malignancy and the second leading cause of cancer deaths worldwide. There were an estimated 1.9 million cases in 2020, with 0.9 million deaths worldwide [1]. Managing this global health burden mandates widespread screening for early detection as well as treatment, which is primarily surgical. The description of laparoscopic colectomy by Jacobs et al. led to a new era of minimally invasive colorectal surgery [2]. This was further augmented by the introduction of the robotic system, da Vinci Surgical System® (Intuitive Surgical, Sunnyvale, CA, USA). Various studies and meta-analyses have compared laparoscopic with open colectomies to establish similar safety and oncological outcomes [3,4]. Laparoscopic surgery has several disadvantages including its learning curve, its fulcrum effect, and a limited degree of freedom. Robotic surgery offers significant improvement in this field with its intuitive wrist system, three-dimensional (3D) viewing, and a high degree of freedom and access. However, it came with the drawback of increased cost. Newer robotic systems are now in the trial stage, and this may alleviate this drawback in the future.

Several trials and observational studies have been done on robotic colorectal resection for colorectal malignancies. Concerning intra-operative outcomes, the duration of surgery was found to be significantly longer in robotic surgery by two meta-analyses [5,6]. These meta-analyses also found a significant reduction in intra-operative conversion rates in robotic surgeries. Pathological outcomes including the number of lymph nodes retrieved, circumferential resection margin, and quality of total mesorectal excision were found to be similar in the landmark “Robotic-Assisted vs Conventional Laparoscopic Surgery on Risk of Conversion to Open Laparotomy Among Patients Undergoing Resection for Rectal Cancer” (ROLARR) trial [7]. These results were re-affirmed by retrospective observational studies [8-10]. There is a paucity of studies comparing long-term oncological outcomes due to the relatively recent nature of robotic surgery. Some studies have reported a similar overall survival and three-year disease-free survival rate. Five-year disease-free survival rate reported by a prospective study was similar for both robotic and laparoscopic methods [10]. This systematic review and meta-analysis aims to perform a critical analysis of available literature and compares non-oncological outcomes following robotic versus laparoscopic colorectal resection for colorectal malignancy.

## Review

Methods

We performed a comprehensive literature search according to the Preferred Reporting Items for Systematic Reviews and Meta-Analyses (PRISMA) guidelines [11]. Electronic databases such as PubMed, PubMed Central (PMC), Medline, and Google Scholar were systematically searched using terms of Medical Subject Headings (MeSH) and keywords up to April 10, 2022. The following search strategy was used, as shown in Table 1, to find relevant studies. The reference lists of selected articles were also examined manually.

**Table 1:** Search Strategy for Electronic Databases

Search Strategy	Database
(("Colectomy/adverse effects"[Majr]) OR ("Colorectal Surgery/adverse effects"[Mesh]) OR outcomes OR ("Robotic Surgical Procedures/adverse effects"[Mesh]) OR ("Laparoscopy/adverse effects"[Mesh])) AND (Robotic colectomy OR laparoscopic colectomy OR robotic colon resection OR laparoscopic colon resection OR robotic colorectal resection OR laparoscopic colorectal resection OR robotic hemicolectomy OR laparoscopic hemicolectomy) AND (Colorectal cancer OR colorectal neoplasm OR colorectal carcinoma OR colorectal malignancy OR colon cancer OR colon neoplasm OR colon carcinoma OR colon malignancy OR rectal cancer OR rectal neoplasm OR rectal carcinoma OR rectal malignancy OR ("Colorectal Neoplasms/surgery"[Majr]))	PubMed, PubMed Central (PMC), and Medline
“Laparoscopic colectomy”, “robotic colectomy”, “colorectal neoplasm”, “outcomes”, and “adverse effects” separately and in combination	Google Scholar

Inclusion Criteria

All studies conducted on humans older than 18 years, published from 2012 to 2022 in the English language, were included.

Exclusion Criteria

Gray literature, books, letters to the editors, case articles, and case series were excluded. Studies not in English and animal studies were also excluded.

Quality Assessment

The following means were used for quality appraisal of the studies: Newcastle Ottawa scale for case-control, cohort, and nonrandomized trials; and Cochrane risk of bias tool (RoB2) for randomized controlled trials. A benchmark of seven stars or "low risk of bias" was set to qualify for our review. Only studies that did colorectal resection for biopsy-proven malignancy were included. Resections for other benign pathologies such as adenoma, ischemia, and emergency surgeries for malignancy complications were excluded. Studies published by the same author and studies utilizing national databases were considered duplicates and excluded. Studies utilizing institute databases were permitted. Data from eligible studies were extracted for various non-oncological postoperative outcomes.

Outcomes of Interest

Primary outcomes were related to recovery post-surgery and included length of hospital stay, time to pass flatus, and time to consume a soft diet. Secondary outcomes were anastomotic leakage, intra-abdominal abscess, mechanical obstruction, postoperative ileus, wound infections, readmission rate, postoperative mortality at 30 days, sexual dysfunction, and wound dehiscence or incisional hernia.

Statistical Analysis

For continuous variables, the standardized mean differences (SMDs) and 95% confidence intervals (CIs) were provided by the inverse variance method. For dichotomous outcomes, the risk ratios (RRs) and 95% CI were calculated by the Mantel-Haenszel model. All results were displayed in forest plots. The I^2^ was reported as a statistical measure of heterogeneity, and if found more than 50%, data were considered heterogeneous and random-effects model was used instead of fixed-effects model. A P < 0.05 was considered significant. The data analysis was performed using the meta-analysis software Review Manager (RevMan) v 5.4.1 (Copenhagen: The Nordic Cochrane Centre, The Cochrane Collaboration, 2020) [12].

Results

Study Selection

**Figure 1:**
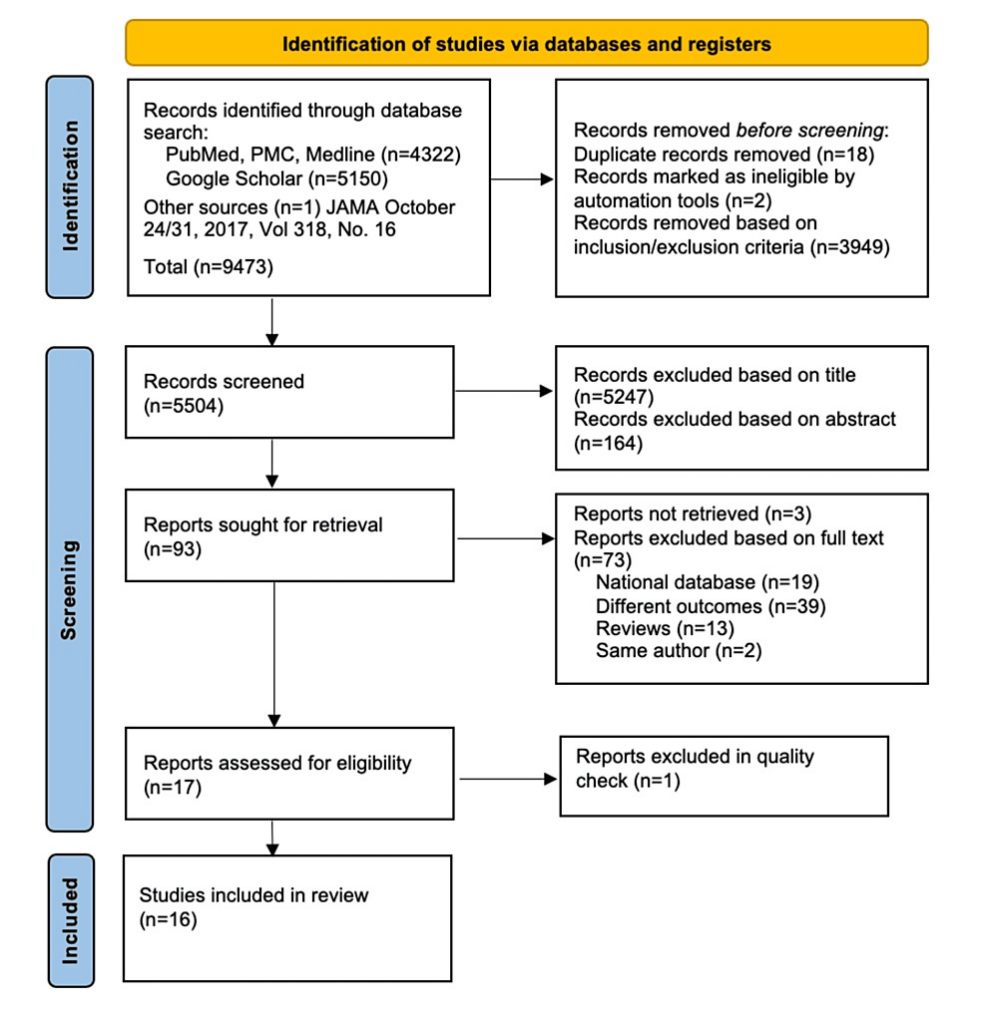
PRISMA Flowchart Preferred Reporting Items for Systematic Reviews and Meta-Analyses (PRISMA) 2020 flowchart showing study selection [11]. PMC: PubMed Central.

A total of 9,473 studies were identified through database search as shown in Figure 1. After the exclusion of duplicates, articles were selected by inclusion and exclusion criteria. A total of 5,504 articles were then screened by title and abstract. Ninety-three articles were retrieved for full text. Three could not be retrieved. Articles with different outcomes and utilizing the same patient database were excluded. Seventeen studies then underwent quality appraisal to finalize the articles for review.

Study Characteristics

Our study included 2,318 patients from 16 studies [7,8,13-26]. Among these studies, six were from South Korea, three from Italy, and one each from France, Germany, Ireland, Spain, Taiwan, and Turkey. One trial was a multinational, multicentric trial from the United Kingdom, Italy, Denmark, the United States, Finland, South Korea, Germany, France, Australia, and Singapore. Studies selected had matched data for the age and sex of participants or had no statistically significant difference between them. Apart from this, two trials were randomized controlled trials, and four had propensity-matched subjects in their studies. Table 2 summarizes the studies included.

**Table 2:** Studies Selected and Their Characteristics

No	Study	Year	Country	Type of Study	Total Patients Laparoscopic Access	Total Patients Robotic Access
1	Ceccarelli et al. [13]	2021	Italy	Propensity-matched retrospective cohort	20	20
2	Fleming et al. [23]	2021	Ireland	Propensity-matched cohort	64	64
3	Galata et al. [8]	2019	Germany	Prospective cohort	33	18
4	Park et al. [14]	2019	South Korea	Randomized controlled trial	35	35
5	de'Angelis et al. [15]	2018	France	Propensity-matched prospective cohort	43	43
6	Kim et al. [16]	2018	South Korea	Prospective nonrandom trial	51	20
7	Jayne et al. [7]	2017	The United Kingdom, Italy, Denmark, The United States, Finland, South Korea, Germany, France, Australia, and Singapore	Randomized controlled trial	230	236
8	Huang et al. [17]	2017	Taiwan	Prospective nonrandom trial	38	40
9	Ielpo et al. [18]	2017	Spain	Prospective observational	112	86
10	Ferrara et al. [19]	2016	Italy	Retrospective cohort	58	42
11	Kim et al. [20]	2016	South Korea	Prospective matched cohort	66	33
12	Cho et al. [21]	2015	South Korea	Propensity-matched retrospective cohort	278	278
13	Guerrieri et al. [25]	2015	Italy	Retrospective observational	23	24
14	Baek et al. [26]	2013	South Korea	Retrospective cohort	37	47
15	Erguner et al. [24]	2013	Turkey	Retrospective cohort	37	27
16	Lim et al. [22]	2013	South Korea	Retrospective observational	146	34

Primary Outcomes

**Figure 2:**
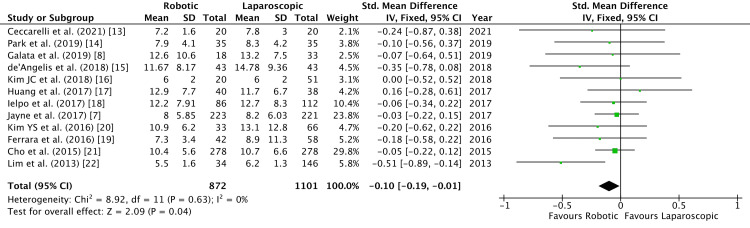
Forest Plot of Comparison: Length of Hospital Stay IV: inverse variance method; Chi^2^: Chi-squared test; df: degree of freedom; I^2^: I^2 ^test for heterogeneity; Z: standard score.

Length of hospital stay was reported by 12 studies as a mean with standard deviation (SD) [7,8,13-22]. Two other studies reported it as median and inter-quartile range. To improve statistical analysis, they were excluded [23,24]. Another study reported incomplete data for analysis [26]. A significant difference was determined in the length of stay between the robotic and laparoscopic groups (SMD = -0.10, 95% CI = -0.19, -0.01, P = 0.04, I^2 ^= 0%), with a shorter duration in the robotic group as shown in Figure 2. 

**Figure 3:**
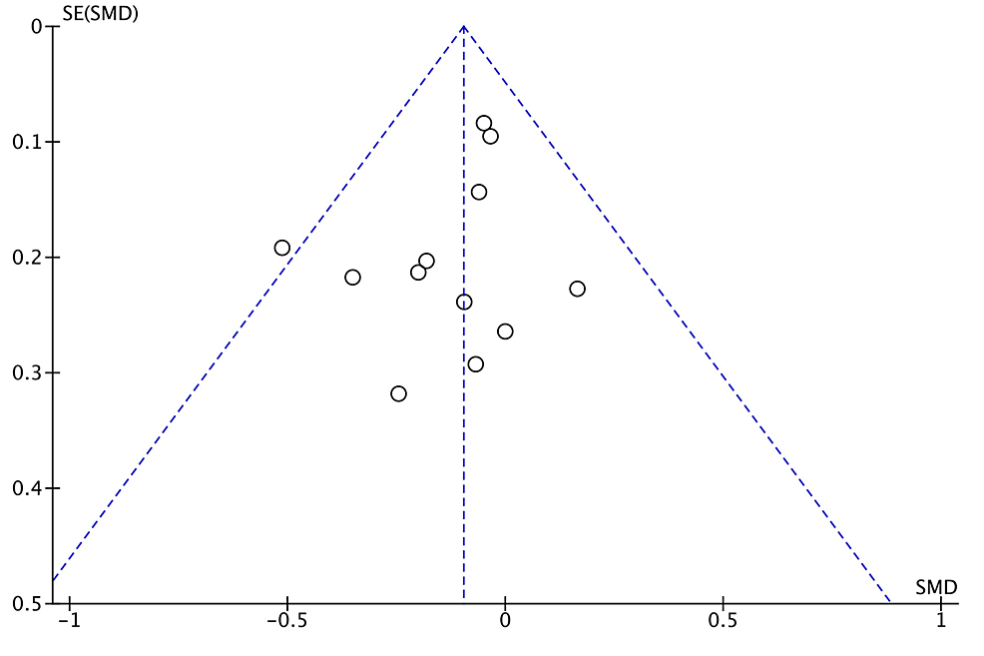
Funnel Plot: Length of Hospital Stay SMD: standardized mean difference.

Funnel plot for the length of hospital stay is shown in Figure 3.

**Figure 4:**
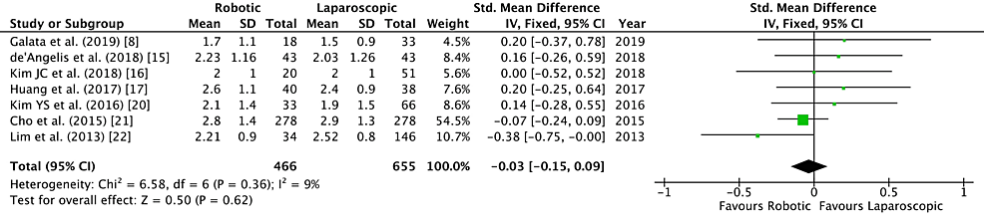
Forest Plot of Comparison: Time to Flatus IV: inverse variance method; Chi^2^: Chi-squared test; df: degree of freedom; I^2:^ I^2^ test for heterogeneity; Z: standard score.

Seven studies observed the time taken to pass flatus postoperatively [8,15-17,20-22]. There was no difference between the two groups (SMD = -0.03, 95% CI = -0.15, -0.09, P = 0.62, I^2 ^= 9%) (Figure 4).

**Figure 5:**
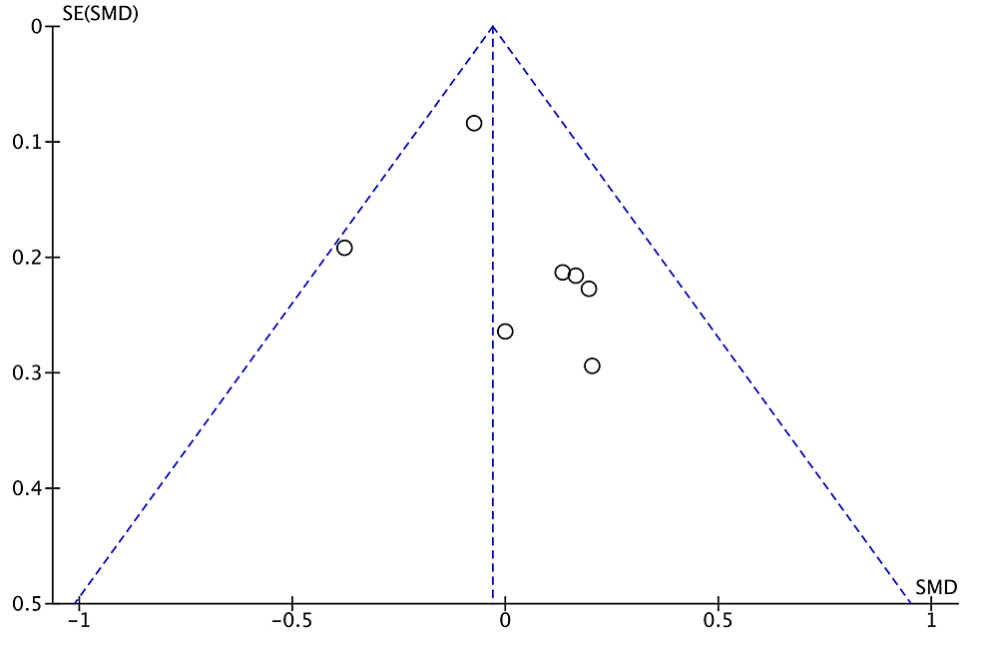
Funnel Plot: Time to Flatus SMD: standardized mean difference.

Figure 5 shows funnel plot for time taken to pass flatus. 

**Figure 6:**
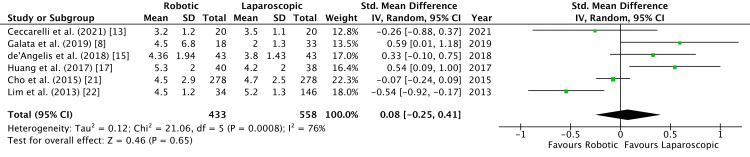
Forest Plot of Comparison: Time to Soft Diet IV: inverse variance method; τ^2^: τ-squared test for random effects model; Chi^2^: Chi-squared test; df: degree of freedom; I^2^: I^2^ test for heterogeneity; Z: standard score.

Time taken to consume soft diet was measured by six studies [8,13,15,17,21,22]. No significant difference was found in the random-effects model (SMD = -0.08, 95% CI = -0.25, 0.41, τ^2 ^= 0.12, P = 0.65, I^2 ^= 76%) (Figure 6).

**Figure 7:**
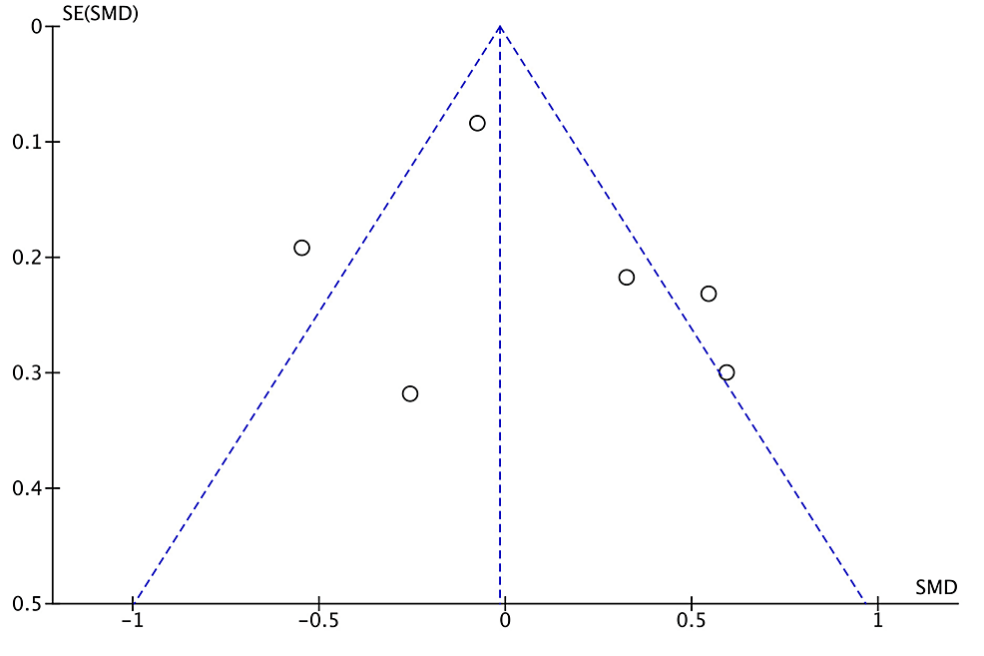
Funnel Plot: Time to Soft Diet SMD: standardized mean difference.

Figure 7 shows funnel plot for time taken to consume soft diet. 

Secondary Outcomes

**Figure 8:**
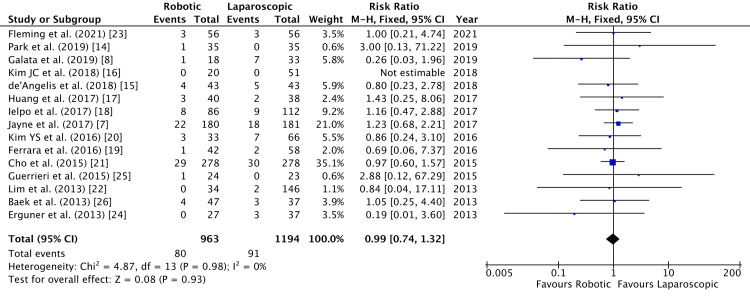
Forest Plot of Comparison: Anastomotic Leakage M-H: Mantel-Haenszel test; Chi^2^: Chi-squared test; df: degree of freedom; I^2^: I^2^ test for heterogeneity; Z: standard score.

Anastomotic leakage was an outcome in 15 studies [7,8,14-26]. No significant difference existed between the robotic and laparoscopic groups (RR = 0.99, 95% CI = 0.74, 1.32, P = 0.93, I^2 ^= 0%) as shown in Figure 8.

**Figure 9:**
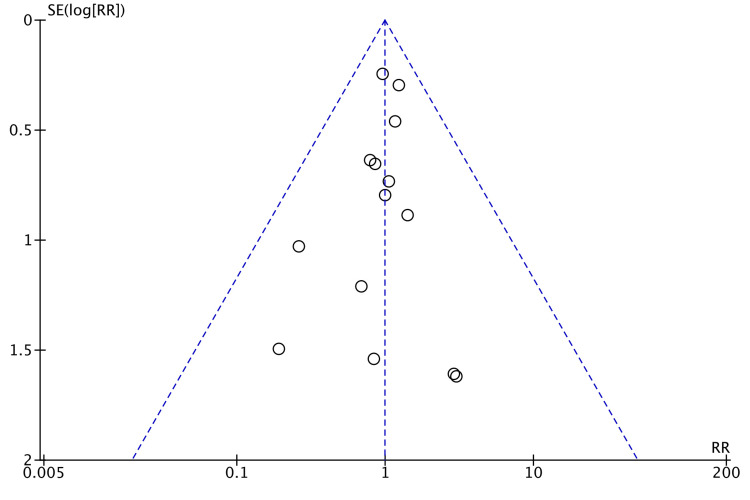
Funnel Plot: Anastomotic Leakage RR: risk ratio.

Funnel plot for anastomotic leakage is shown in Figure 9. 

**Figure 10:**
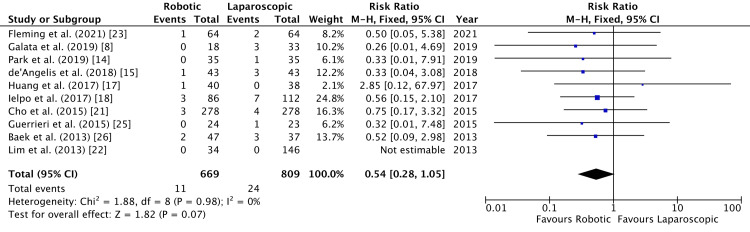
Forest Plot of Comparison: Intra-Abdominal Abscess M-H: Mantel-Haenszel test; Chi^2^: Chi-squared test; df: degree of freedom; I^2^: I^2^ test for heterogeneity; Z: standard score.

Ten studies compared the incidence of intra-abdominal abscess postoperatively [8,14,15,17,18,21-23,25,26]. While there was a slightly higher incidence after laparoscopic surgery, it was not statistically significant (RR = 0.54, 95% CI = 0.28, 1.05, P = 0.07, I^2 ^= 0%) (Figure 10).

**Figure 11:**
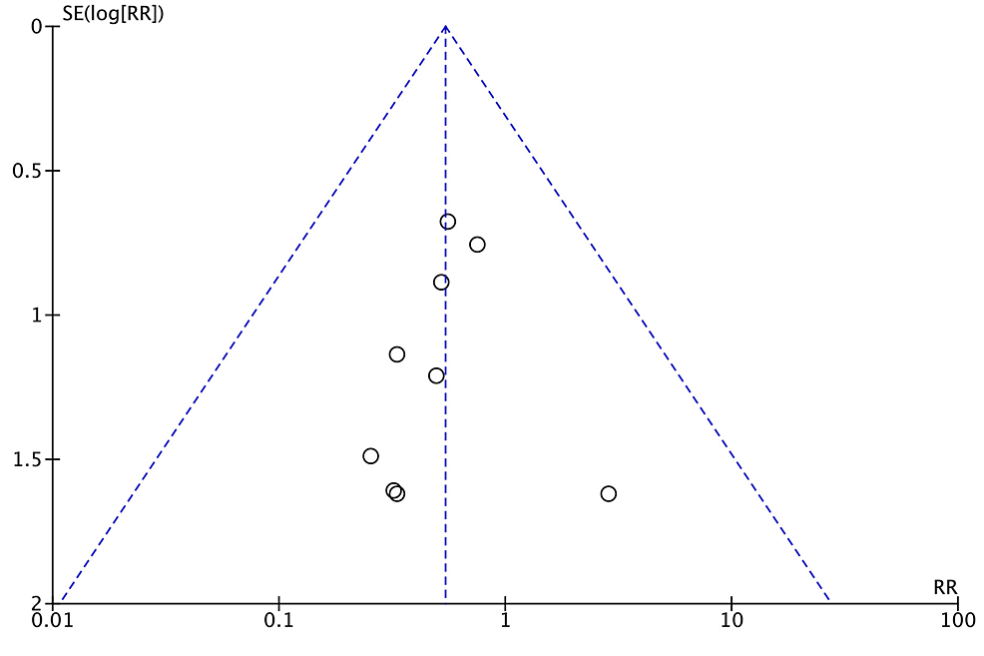
Funnel Plot: Intra-Abdominal Abscess RR: risk ratio.

Funnel plot for intra-abdominal abscess is shown in Figure 11.

**Figure 12:**
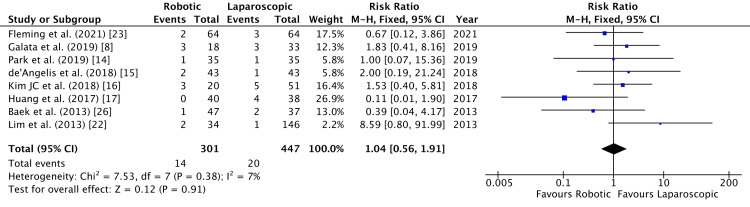
Forest Plot of Comparison: Postoperative Ileus M-H: Mantel-Haenszel test; Chi^2^: Chi-squared test; df: degree of freedom; I^2^: I^2^ test for heterogeneity; Z: standard score.

Postoperative ileus was compared in eight studies [8,14-17,22,23,26]. Figure 12 shows that no difference was observed between robotic and laparoscopic surgeries (RR = 1.04, 95% CI = 0.56, 1.91, P = 0.91, I^2 ^= 7%).

**Figure 13:**
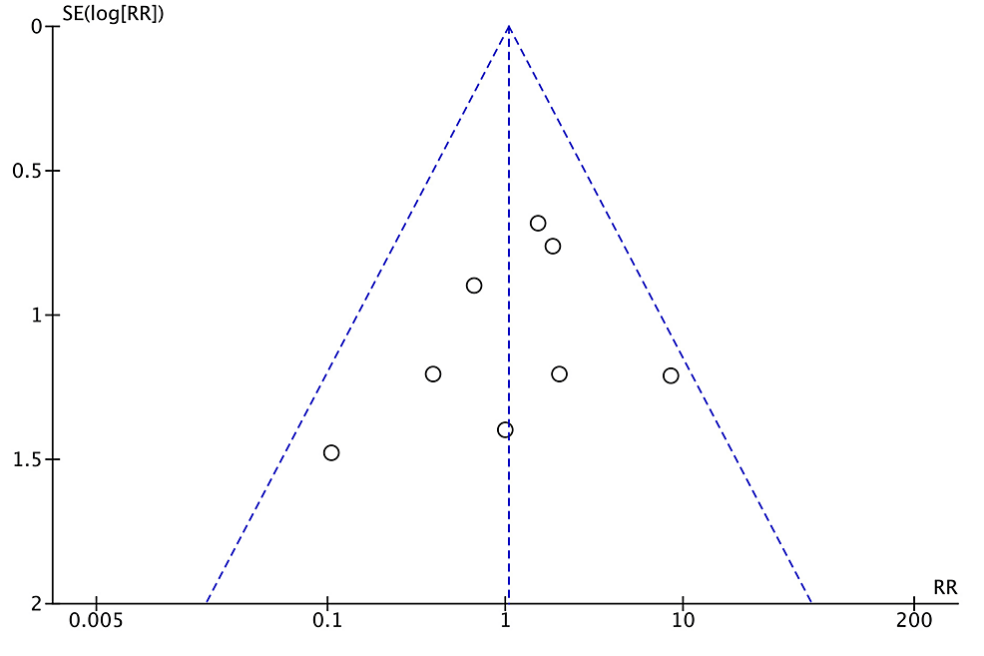
Funnel Plot: Postoperative Ileus RR: risk ratio.

Figure 13 shows funnel plot for postoperative ileus. 

**Figure 14:**
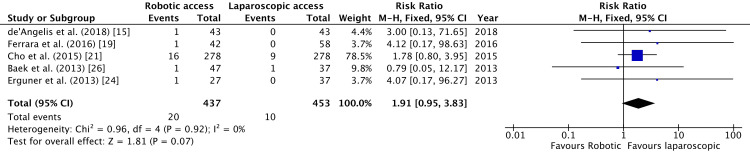
Forest Plot of Comparison: Mechanical Obstruction M-H: Mantel-Haenszel test; Chi^2^: Chi-squared test; df: degree of freedom; I^2^: I^2^ test for heterogeneity; Z: standard score.

Five studies reported mechanical obstruction after surgery [15,19,21,24,26]. Robotic surgery caused a higher incidence of postoperative mechanical obstruction in the studies observed (RR = 1.91, 95% CI = 0.95, 3.83, P = 0.07, I^2 ^= 0%), but this was not statistically significant (Figure 14).

**Figure 15:**
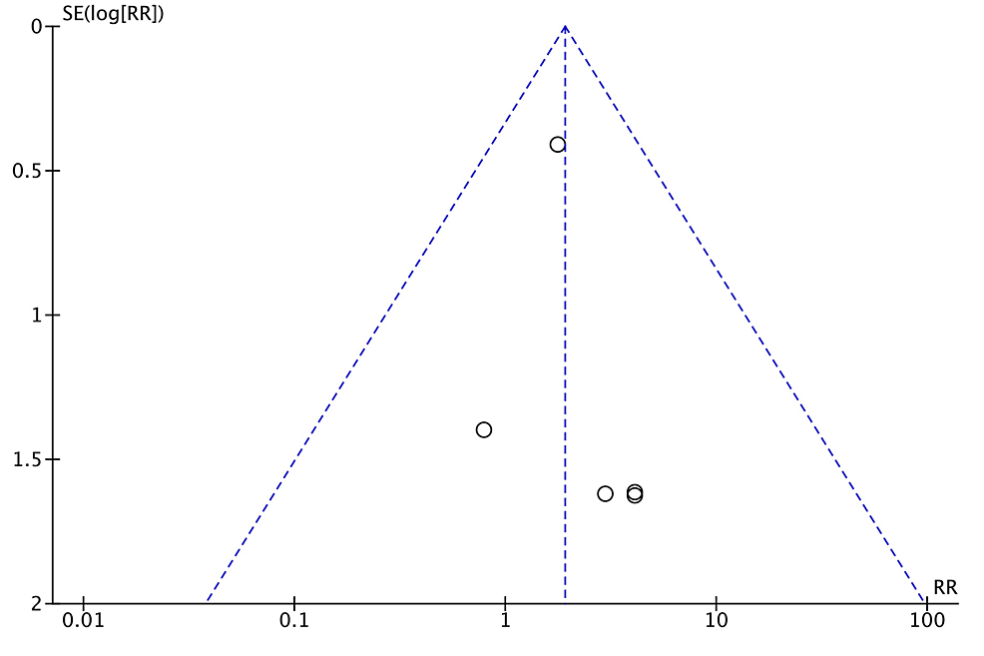
Funnel Plot: Mechanical Obstruction RR: risk ratio.

Figure 15 shows funnel plot for mechanical obstruction. 

**Figure 16:**
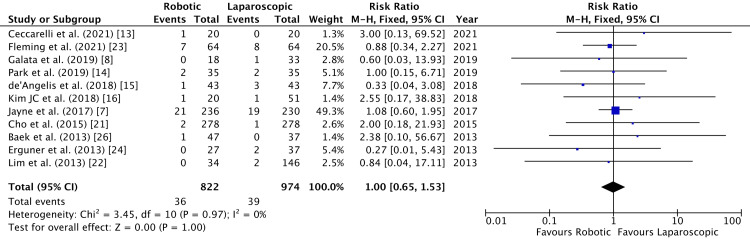
Forest Plot of Comparison: Wound Infection M-H: Mantel-Haenszel test; Chi^2^: Chi-squared test; df: degree of freedom; I^2^: I^2^ test for heterogeneity; Z: standard score.

Wound infection was described by 11 studies [7,8,13-16,21-24,26]. The incidence in both robotic and laparoscopic arms of study was similar (RR = 1.00, 95% CI = 0.65, 1.53, P = 1.00, I^2 ^= 0%) (Figure 16).

**Figure 17:**
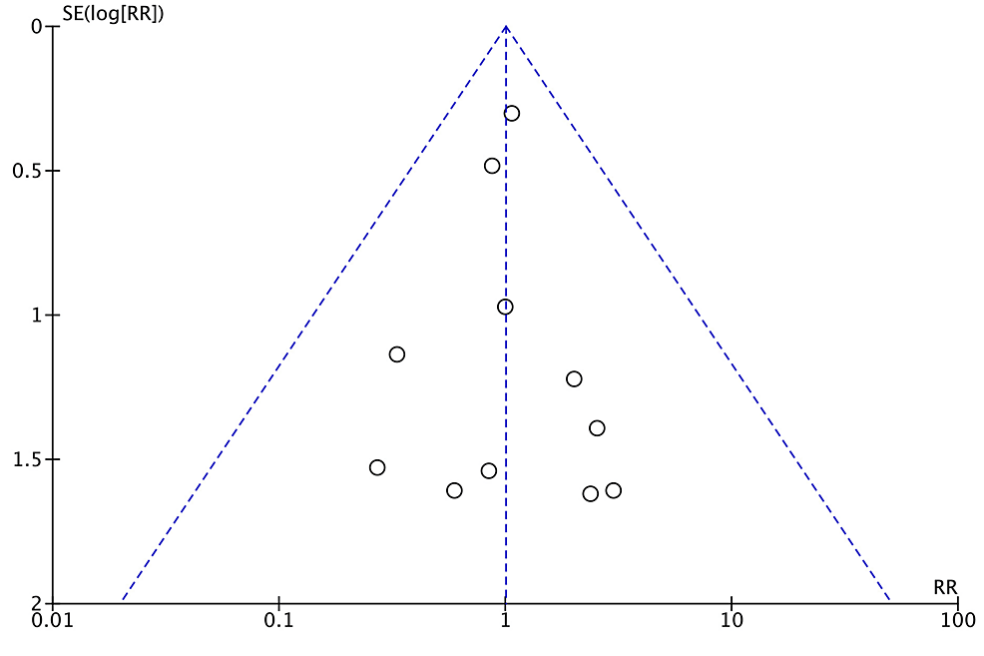
Funnel Plot: Wound Infection RR: risk ratio.

Funnel plot for wound infection is shown in Figure 17.

**Figure 18:**
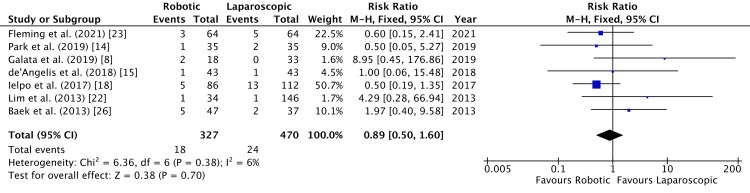
Forest Plot of Comparison: Readmission M-H: Mantel-Haenszel test; Chi^2^: Chi-squared test; df: degree of freedom; I^2^: I^2^ test for heterogeneity; Z: standard score.

Seven studies reported readmission [8,14,15,18,22,23,26]. No significant difference was observed between the two groups (RR = 0.89, 95% CI = 0.50,1.60, P = 0.7, I^2 ^= 6%) as shown in Figure 18.

**Figure 19:**
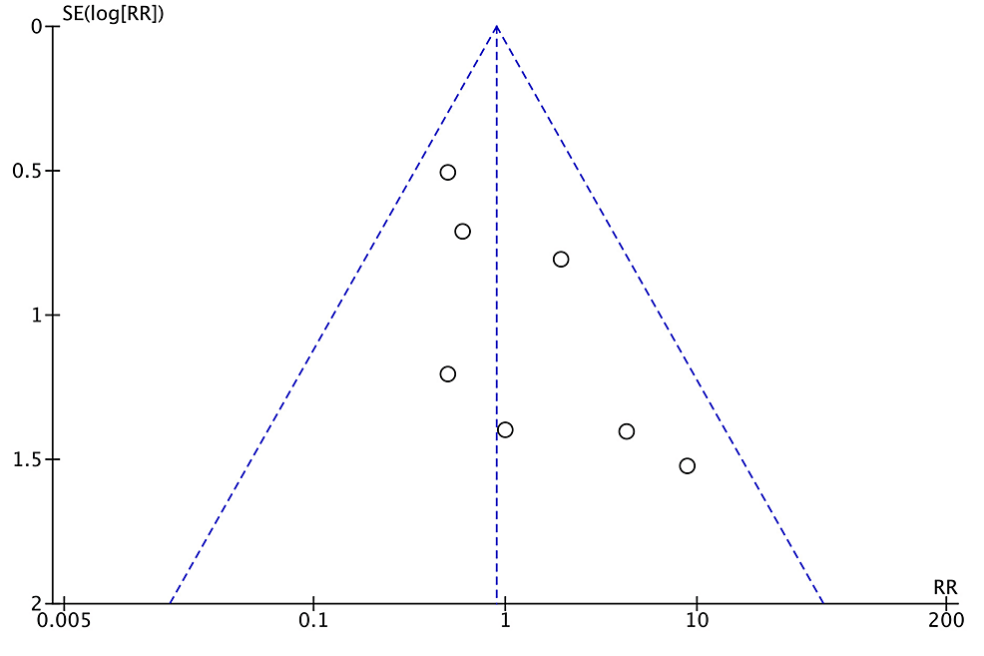
Funnel Plot: Readmission RR: risk ratio.

Funnel plot for readmission is shown in Figure 19.

**Figure 20:**
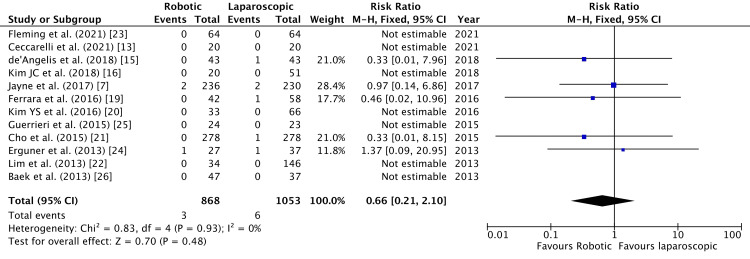
Forest Plot of Comparison: Postoperative Mortality M-H: Mantel-Haenszel test; Chi^2^: Chi-squared test; df: degree of freedom; I^2^: I^2^ test for heterogeneity; Z: standard score.

Mortality in 30 days was reported by 11 studies [7,13,16,19-26]. One study reported mortality in 90 days [15]. Mortality was higher in laparoscopic surgery, but not statistically significant (RR = 0.66, 95% CI = 0.21, 2.10, P = 0.48, I^2 ^= 0%) (Figure 20).

**Figure 21:**
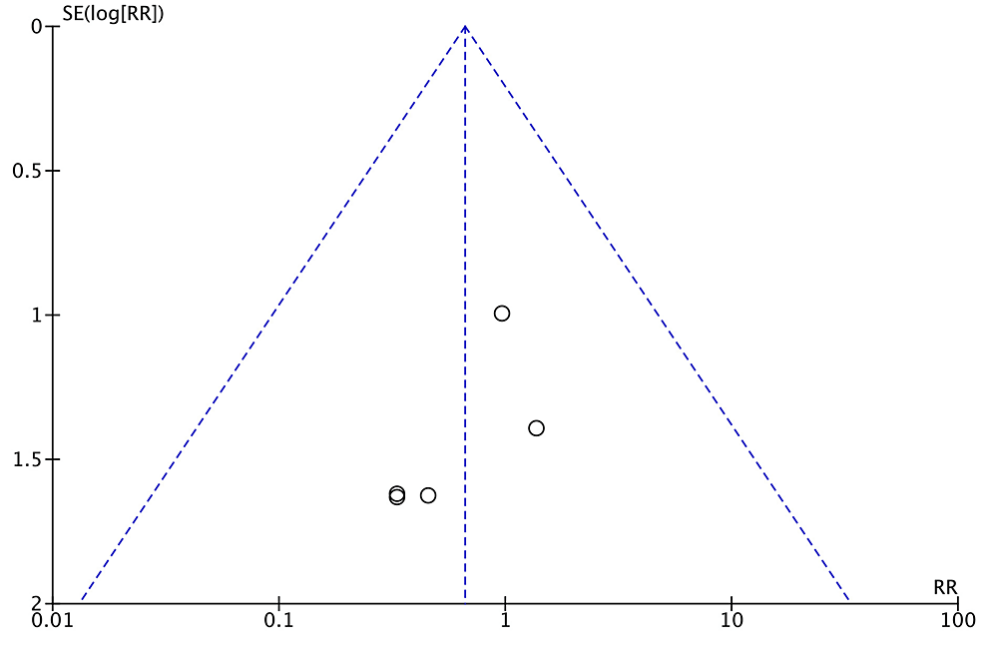
Funnel Plot: Postoperative Mortality RR: risk ratio.

Figure 21 shows funnel plot for postoperative mortality. 

**Figure 22:**
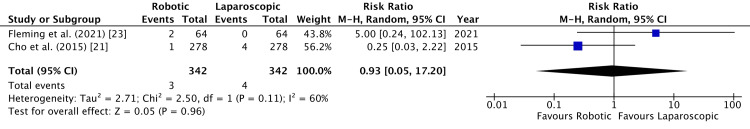
Forest Plot of Comparison: Postoperative Wound Dehiscence or Hernia M-H: Mantel-Haenszel test; τ^2^: τ-squared test for random effects model; Chi^2^: Chi-squared test; df: degree of freedom; I^2^: I^2^ test for heterogeneity; Z: standard score.

Two studies followed up patients to observe wound dehiscence or hernia [21,23]. Incidence was similar following robotic and laparoscopic surgeries using random-effects model (RR = 0.93, 95% CI = 0.05, 17.20, τ^2 ^= 2.71, P = 0.96, I^2 ^= 60%) (Figure 22).

**Figure 23:**
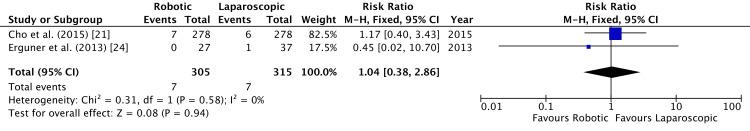
Forest Plot of Comparison: Sexual Dysfunction M-H: Mantel-Haenszel test; Chi^2^: Chi-squared test; df: degree of freedom; I^2^: I^2^ test for heterogeneity; Z: standard score.

Sexual dysfunction was quantified differently in different studies. Two studies mentioned the number of events of sexual dysfunction [21,24]. No difference was found on analyzing data for the two groups (RR = 1.04, 95% CI = 0.38, 2.86, P = 0.94, I^2 ^= 0%) (Figure 23).

Discussion

Currently, the decision to undergo laparoscopic or robotic surgery is often made by patient choice and their affordability. Availability of the robotic system and access to services is another factor. A high-volume center and surgeon expertise can also make a huge difference in the outcomes reported. Some studies have attempted to chart the number of surgeries required to define the learning curve of laparoscopic and robotic surgeries. A review by Pernar et al. concludes that, for colorectal surgery, 15 to 25 cases are needed to master the initial learning curve, with expertise attained at 75 to 128 cases [27]. Another important factor that could potentially alter outcomes is surgeon distress while performing the procedure. An article published by the Mayo Clinic compared surgeon workload during robotic, laparoscopic, and open surgery [28]. It suggested that robotic surgery offers the surgeon better control over the field leading to diminished frustration. Laparoscopic surgery, on the other hand, requires more physical effort and mental exertion. Notwithstanding these likely confounding factors, the studies included reported data from surgeries performed by surgeons operating at high-volume centers.

Primary Outcomes

Recovery from colorectal surgery may be quantified by temporal variables such as duration of stay in hospital and time taken for the return of bowel activity. In our analysis, we established a substantial difference in the length of hospital stay, measured in days, between robotic and laparoscopic groups (Figure 2). This differs from the findings of the ROLARR trial, which stated no such disparity [7]. Most of the included papers, while showing a shorter duration of stay, did not display statistical significance. Baek et al. also stated a significant difference in the duration of hospital stay postoperatively, favoring the robotic group (nine vs 11 days, P = 0.01) [26]. Another meta-analysis, by Safiejko et al., also observed a significant difference between the two groups [29]. However, the data were highly heterogeneous (I^2^ = 99%) [29]. A shorter length of hospital stay is associated with enhanced recovery after surgery and diminished financial burden. Pascal et al. evaluated 98,713 patients in 540 hospitals for colectomy and could not find a correlation between shortened length of stay and readmission rates [30].

Bowel recovery was documented by seven studies by recording the time of passing flatus after surgery [8,15-17,20-22]. We observed no significant difference between the two groups of study (Figure 3). Lim et al. noted a significant difference in time to pass stools (3.85 vs 4.42 days, P = 0.003) in their study [22]. Commencement of soft diet after surgery in pooled data indicated no significant difference in robotic and laparoscopic groups (Figure 4). This echoed the findings of the meta-analysis by Safiejko et al. [29]. Robotic surgery has a significantly longer operative time as demonstrated by Kim et al. in a propensity-matched study (P < 0.0001) comprising 224 patients in each arm of the study [31]. The longer duration of surgery by robotic access could be a confounding factor with regard to the functioning of the bowel.

Secondary Outcomes

We analyzed the incidence of different postoperative non-oncological outcomes across the selected studies. All the studies followed up their patients and reported early postoperative outcomes within 30 days of surgery. Anastomotic leakage was deemed to be similar between robotic and laparoscopic groups, with an RR of 0.99 (Figure 5). Walker et al. scrutinized the effect of anastomotic leakage on survival rates [32]. They established a negative effect of anastomotic leak on both overall survival and cancer-specific survival. This highlights that anastomotic leak is detrimental to patients not only in the postoperative period but also in the long run. A slightly higher incidence (RR = 0.54) of intra-abdominal abscess following laparoscopic colorectal resection was established in our meta-analysis. Nevertheless, this was not statistically significant (Figure 6).

Postoperative ileus incidence did not vary between robotic and laparoscopic colorectal resections (Figure 7). A positive correlation was observed by Scheer et al., in their study of 487 patients who had undergone colorectal resection, between the duration of surgery and postoperative ileus [33]. Moreover, a retrospective analysis by Campana et al. investigated laparoscopic right- versus left-sided colectomies in high-volume centers [34]. They concluded that right-sided colectomy for colon cancer had a shorter operative time possibly leading to an increased postoperative ileus and longer hospital stay than left laparoscopic colectomy. More recently, Nasseri et al. established no such differences between right and left colectomies when robotic surgery was done [35]. Our study did not discriminate between different types of colorectal resections. Thus, pooled rates of postoperative ileus may not be representative of differences, if any, between right- and left-sided colectomies.

Mechanical bowel obstruction after colorectal resection was found to be higher in the robotic arm (RR = 1.91). However, this was not statistically significant (Figure 8). Postoperative mechanical bowel obstruction is commonly due to adhesions. Dense, inflammatory adhesions usually form within 10 to 14 days. With minimally invasive surgery, there is reduced trauma to the bowel and other tissues, resulting in fewer intraperitoneal adhesions [36]. Goussous et al. confirmed this in their trial where laparoscopic surgery resulted in fewer adhesive obstructions compared to open surgeries (P < 0.01) [37]. However, they also found that laparoscopic surgery was associated with a higher incidence of strictures (P = 0.03), which can again cause mechanical bowel obstruction. More trials are needed to comment on the nature of mechanical bowel obstruction and its occurrence after laparoscopic and robotic colorectal excisions.

Incidence of wound infection was evaluated to be comparable (RR = 1) (Figure 9). Readmission rate was also found to be similar between the two groups (Figure 10). Readmission within 30 days was considered by some studies [8,22,23]. The others followed up for prolonged readmission for postoperative complications. Ielpo et al. noted readmission within 90 days of surgery and found a significant difference between robotic and laparoscopic groups (P = 0.001) [18]. This was tied to the higher incidence of intra-abdominal abscess in the laparoscopic group, requiring antibiotics or drainage as indicated [18]. Postoperative 30-day mortality was noted by all studies in Figure 11, except by de’Angelis et al. who reported a 90-day mortality rate [15]. Jayne et al. reported two deaths in each group, all due to septic complications due to surgery [7]. Erguner et al. reported a death in the laparoscopic group due to cardiac reasons and in the robotic group due to sepsis [24]. It is reiterated that readmission rates were mostly a result of complications and independent of the initial length of hospital stay [30].

Incisional hernias were reported by two studies [21,23]. While there was no significant difference between robotic and laparoscopic surgeries (Figure 12), more studies are needed with longer follow-up of patients to substantively opine on this matter. A single institute retrospective study comparing 276 patients who underwent robotic or laparoscopic right hemicolectomy inferred a similar rate of incisional hernias (17.4% vs 22.2% robotic vs laparoscopic) after a median follow-up of 9.2 months [38].

Only two studies quantified the absolute number of patients with sexual dysfunction as a result of surgery [21,24]. No significant difference was found between robotic and laparoscopic groups in our analysis (Figure 12). ROLARR trial found no significant difference in male and female sexual dysfunction postoperatively between the two groups [7]. Confirming this, Galata et al. reported a slightly improved male and female sexual function following robotic surgery, but this was again not found statistically significant [8]. Cho et al. argued that voiding dysfunction is significantly higher in laparoscopic group than in robotic group (4.3% vs 0.7% P = 0.012) [21]. Impaired urogenital function remains a massive adverse event following rectal surgery. Nerve injuries involving the inferior hypogastric plexus can cause sexual dysfunction in patients [39]. Improved vision and precision of robotic surgery may be beneficial here. Kim et al. in their comparative study found earlier recovery of voiding and sexual function in patients who underwent robotic total mesorectal excision compared to laparoscopic total mesorectal excision [40]. Luca et al. in their study of 74 patients who underwent robotic rectal surgery commented that sexual satisfaction was comparable after one year of surgery to preoperative level, suggesting preservation of sexual function by robotic surgical dissection [41].

Limitations

The inclusion of nonrandomized and observational studies in the meta-analysis poses a risk of bias. However, four of the included cohort studies had a propensity-matched population in both groups for analysis. The rest of the studies found no difference between the study population with respect to the age and sex of patients. Another limitation of the study is including both colon and rectal surgeries in one group. Surgeons' decision to choose laparoscopic or robotic surgery may have been based on comfort and perceived difficulty of the case. More trials with longer follow-up are needed to document long-term postoperative outcomes such as incisional hernia and bowel obstruction.

## Conclusions

This systematic review and meta-analysis compared the incidence of various non-oncological postoperative outcomes following minimally invasive colorectal resection for colorectal malignancies. A statistically significant difference was noted in the length of postoperative hospital stay, favoring the robotic approach. Intra-abdominal abscess rates were higher following laparoscopic access, whereas mechanical obstruction was higher with robotic access. Time to flatus, time to soft diet, and rates of anastomotic leakage, ileus, wound infection, readmission, mortality, and incisional hernias were similar among the two groups. Urogenital dysfunction, while initially similar, has earlier recovery by robotic approach. Along with the findings of this study, robotic surgery offers an obvious advantage in terms of better vision, greater maneuvering ability and comfort for the surgeon, and a lower conversion rate. Therefore, we can conclude that robotic surgery represents the future of minimally invasive surgery for colorectal cancer.
